# Is there a link between the autonomy of women and maternal healthcare utilization in Nigeria? A cross-sectional survey

**DOI:** 10.1186/s12905-023-02317-z

**Published:** 2023-04-06

**Authors:** Emmanuel Kolawole Odusina, Oluwarotimi Samuel Oladele

**Affiliations:** Department of Demography and Social Statistics, Faculty of Social Sciences, Federal University Oye-Ekiti, Oye-Ekiti, Ekiti State Nigeria

**Keywords:** Maternal health-care, Autonomy of women, Antenatal care visit, Health facility delivery, Postnatal visit, Nigeria

## Abstract

**Background:**

Despite legislation and intervention programmes, the rates of maternal and child mortality in Nigeria remain high. Sustainable development goals on mother and child mortality would be a mirage if this continues. The study investigated the autonomy of women (women’s decision-making autonomy) and the use of maternal health-care services in Nigeria.

**Methods:**

Secondary data obtained from the Nigeria Demographic and Health Survey, 2018 were used in this investigation. Women who indicated they gave birth in the five-year before the surveys were considered in the study. The association between autonomy of women and maternal health-care utilization was studied using binary logistic regression models.

**Results:**

In total, about one-fifth of the women (19.6%) indicated they had at least eight ANC visits for their most recent birth. Overall, 40.5% of the women gave birth in a health institution, and 20.1% went for postnatal checkups. The use of health-care services was significantly related to the autonomy of women. Women’s and husbands/partners’ educational levels, residency and ethnicity were socio-demographic characteristics that influenced women’s healthcare service consumption.

**Conclusions:**

For most recent childbirth, most women did not utilise the health-care services in Nigeria. To enhance the autonomy of women and, as a result, maternal health-care services use in Nigeria, effective interventions, policies, and programmes are required.

## Background

Every year, over 287,000 women in their childbearing age die around the world due to complications during pregnancy and childbirth [1–3]. Despite local, national, regional, and international efforts, programmes, and other policies to curtail maternal and child mortality, nearly all maternal deaths due to complications during pregnancy and childbirth occur in less developed countries [3], with more than two-thirds (about 66%) occurring in sub-Saharan African countries [4]. Maternal mortality has increased in many sub-Saharan African nations [5]. Maternal mortality accounts for about 59,000 deaths annually [5]. Globally, India is ranked first and Nigeria ranked second in maternal incident rate [5, 6]. Nigeria has about 545 prevalence of maternal mortality for every 100, 000 births [5, 7].

Increased usage of health-care services by women and their children is critical for improving women’s health and reducing mother and child morbidity and mortality [8]. In Nigeria, the high rate of women’s death is related to the non-use or poor use of maternal health services [8]. Maternal health-care that is timely, regular, and adequate can help to improve maternal well-being and reduce the risk of negative pregnancy outcomes.

Complications of pregnancy, as well as the mortality associated with them, can be decreased by efforts that increase healthcare delivery, maternal nutrition, and rapid utilization of healthcare facilities whenever there is a need for urgent obstetric care [9]. After a child is born, postnatal health-care services can help to prevent postpartum and life-threatening complications. Without concerted efforts to reduce maternal and child mortality, attaining the Sustainable Development Goals on women and child health would be a mirage [10].

The autonomy of women refers to the power and liberty of women to make decisions about matters that impact them and their families on their own or in collaboration with their partners [8]. They are involved in family decision-making, have access to funds or resources, and partake in resource management and decision-making [11–13]. The roles of women in decision-making have been linked to access and the usage of health-care services [14–16]. Furthermore, women’s status has an impact on women’s and child health [11, 17].

In Nigeria, this study looked into the relationship between the autonomy of women and maternal health-care services utilization (antenatal care (ANC) visitations, delivery at health facility, and postnatal health services) using the most recent Nigeria Demographic and Health Survey dataset. Men in Nigeria have a greater social rank than women. Most family heads, breadwinners, and older partners are men [18]. The patriarchal nature of most Nigerian households, which is reinforced by religion, may have an impact on the autonomy of women and the use of healthcare services, as well as maternal and childhood illness and death. Studies have explored socio-demographic and behavioural issues regarding maternal health-care services utilization. Socio-demographic and behavioural factors such as wealth status, maternal occupation, parity, media exposure were found to be associated with maternal health-care services utilization. Other factors found to be associated with maternal health-care services utilization were complications during pregnancy and antenatal checkups by skilled birth attendants [19–22]. Also, studies abound on timing of first ANC visits, early initiation of ANC visits, inequalities in early ANC visits and factors associated with them. Education, occupation, residence, parity, distance, wealth status and knowledge of the timing of ANC were found to be associated with early initiation of ANC [23–26]. Despite the previous study on the autonomy of women and maternal health-care service utilisation in Nigeria [10, 27–35], the association between maternal health-care services use and the autonomy of women needs further exploration [36]. The high level of maternal morbidity and mortality in Nigeria is an indication of the need for more evidence-based research on maternal health-care services utilization [37]. Studies have been carried out using a composite index of 4 or more ANC visits [38–40], delivery at the health facility, and postnatal care among childbearing women [39, 41]. World Health Organisation recommended a minimum of 8 antenatal care visits for positive pregnancy outcome and to promote the health of mothers [42]. Compliance with the recommended standard of minimum of 8 ANC contacts could improve the indicators of maternal and child health [37]. This study considered a composite index of 8 or more ANC visits, delivery at the health facility, and postnatal care among childbearing women in Nigeria.

Few studies have been conducted recently on autonomy of women and maternal health-care services utilization in Nigeria [10, 34, 35]. Available studies have not fully examined the relationship between the autonomy of women and maternal health-care services utilization in Nigeria particularly the use of a composite index of 8 or more ANC visits along with delivery at the health facility, and postnatal care services [10]. Examination of the link between the autonomy of women and maternal health-care services utilization using a recent dataset is important to address issues of maternal and child needs in Nigeria. As a result, the purpose of this study is to investigate the relationship between the autonomy of women and the use of maternal health-care services in Nigeria. This is required because mother and child mortality are high, and maternal health-care resources are underutilized [43]. The findings of this study will enhance understanding of the relationship between the autonomy of women and the use of maternal health-care services, as well as the factors that influence these relationships. It will also drive policies and intervention programs on the usage of maternal health services, potentially lowering the country’s mother and child morbidity and death rates.

## Methods

### Source of data

Data from the Nigeria Demographic and Health Survey (NDHS), a cross-sectional, national, and representative sample survey, were used in the study. The information was acquired under the supervision of the National Population Commission, which oversees such matters. The survey’s goal was to gather current and trustworthy information on family planning, fertility, HIV/AIDS knowledge, domestic violence, nutritional status of children, maternal health, child health and health-care services utilization. The Nigeria Population Commission and the ICF Macro Institutional Review Board in Calverton, Maryland, USA, both accepted the study’s protocol. There is no need for independent ethical approval because the dataset is freely available on the official DHS website [44].

### Study participants

The survey included married women of age group 15–49 who indicated they gave birth in the five years prior to the survey (n = 19,764). Detailed information on the questionnaire and methods used to conduct the survey can be found somewhere [7].

### Outcome variable

The outcome variable is maternal health-care services utilization (a composite index of 8 or more ANC visits [42], delivery at health facility, and postnatal care of a recent or current child born within the five-year before the survey). ANC visits was coded as one if a respondent reported eight or more ANC visits for the latest childbirth, otherwise zero. Health facility delivery was coded as one if a respondent used health facility at childbirth for the latest childbirth, otherwise zero. Postnatal care was coded as one if a respondent went for postnatal care services for the latest childbirth, otherwise zero.

### Independent variables

The autonomy of women was one of the independent variables studied (the composite index of decision-making on health-care services for respondents, huge household purchases and visitations to relatives or family) [8]. Other covariates included education (no formal education, primary education, and post or higher secondary education), ethnicity (Yoruba, Igbo, Hausa/Fulani and others), children ever born, respondents’ age (<25, 25–34, and 35+), partners’ age (<25, 25–34, and 35–44, and 45+), level of education of partners or spouses (no formal form education, primary education, and post or higher secondary education), wealth index (rich, middle and poor), residence (urban and rural) and religion (Islam and Christianity). To account for sampling variability, sample weight was used [8].

### Data analysis

The study employed three types of analyses: univariate, bivariate, and multivariate. The frequency and percentage distributions of variables were explored in the univariate analysis. Using the Chi-square test, the bivariate analysis looked at the relationship between a dependent (maternal health-care services utilization - antenatal care (ANC) visitations, delivery at health facility, and postnatal health services) and independent variables (autonomy of women, education, and age of respondent and partner, wealth status, residence, religion, ethnicity, and children ever born). In addition, a test of correlation was performed among explanatory variables to account for multicollinearity. The results of the test demonstrated that the multicollinearity assumptions were not violated. More than 0.10 was the tolerance value [45]. The multivariate analysis explored the influence of the autonomy of women on maternal health-care use using logistic regression models.

## Results

Table 1 presents the descriptive features of the participants. The study included a weighted sample of 19,764 married participants who gave birth in five-year preceding the survey. Almost two-fifths of respondents (38.6%) indicated they were not involved in decisions that affected their lives and households. Approximately 25% of the mothers were under the age of twenty-five, and almost two-fifth of the mothers (38.2%) was between the ages of 25 and 34. More than one-third of the women (47.3%) and their spouses (38.2%) reported no formal form of education, and more than a quarter of the women (29.6%) and their spouses (32.9%) had secondary education. Muslim women made up a higher number of married women (64.6%), while 62.0% of married women lived in rural regions. The Hausa/Fulani ethnic group accounted for 49.8% of the total population. A higher percentage of those polled had given birth to four or more children.

**Table 1:** Features of the sampled population

Variables	Number	Percentage
**Women’s Autonomy**		
Not involved	7627	38.6
Involved	12,137	61.4
**Age Group**		
< 25	4768	24.1
25–34	9529	48.2
35+	5467	27.7
**Education Level**		
No Formal Form of Education	9356	47.3
Primary Level of Education	2852	14.4
Secondary Level of Education	5848	29.6
Post Secondary Level of Education	1708	8.6
**Religion**		
Christianity	6997	35.4
Islam	12,767	64.6
**Wealth Index**		
Poor	8976	45.4
Middle	3980	20.1
Rich	6808	34.5
**Residence**		
Urban	7511	38.0
Rural	12,253	62.0
**Ethnicity**		
Hausa/Fulani	9849	49.8
Igbo	2353	11.9
Yoruba	2218	11.2
Others	5343	27.0
**CEB**		
One	3076	15.6
Two	3549	18.0
Three	3019	15.3
Four plus	10,120	51.2
**Age Group of Partners**		
< 25	357	1.81
25–34	5238	26.5
35–44	7908	40.0
45+	6260	31.7
**Level of Education of Husbands/Partners**		
No Formal Form Education	7554	38.2
Primary Level of Education	2726	13.8
Secondary Level of Education	6499	32.9
Post Level of Secondary	2985	15.1
Total	19,764	100.0

Respondents’ utilization of maternal health-care services is presented in Table 2. In the five-year preceding the survey, less than one-fifth of the mothers (19.6%) who had a child had 8 or more prenatal care visits. About 40.5% of the mothers had their last child in a health institution, and about one-fifth of the women (20.1%) claimed they went for postnatal care. In the five-year prior to the study, less than 41.0% of the mothers used all three services for their previous live birth (8 or more ANC checkups, health facility delivery as at last birth, and postnatal visits).

**Table 2:** Utilization of maternal health-care services in five-year preceding the survey

Variables	Number	Percentage
ANC Visits		
< 8 Visits	15,887	80.4
8 or More Visits	3876	19.6
**Place of Delivery**		
Delivery at Home	11,755	59.5
Delivery at Health Facility	8008	40.5
**Postnatal Check UP**		
No	15,798	79.9
Yes	3966	20.1

Table 3 shows the background and other selected variables by maternal health-care services utilization for the most recent or current live birth in the five-year prior to the survey. When compared to women who claimed they did not have autonomy in decision-making, mothers (7.8%) who indicated they did had autonomy in decision-making used more maternal health-care services (ANC visits, 26.2%; health facility delivery, 50.3% and postnatal care services, 24.9%). In comparison to mothers under the age of 25, mothers above the age of 25 used maternal healthcare services more frequently. The utilization of maternal health-care services rose with an increase in education; respondents with at least a secondary level of education used maternal health-care services more than respondents with only a primary level of education. Christian women accessed maternity health-care more frequently than Muslim women. Respondents from wealthy households, those who lived in cities, and those who belonged to the Yoruba tribe and had one child used maternal healthcare services more frequently. The usage of maternal health-care services by women increases with the education of husbands/partners. In comparison to males in other age groups, wives of men in the 35–44 age group used maternal health-care services more. As presented in Table 3, the autonomy of women and all the background features (age, education, religion, wealth index, residence, region, children ever born (CEB) and ethnicity) of women were statistically associated with maternal health-care services utilization.

**Table 3:** Background features of women by maternal health-care services use

Variables	N	ANC Visits	P-value	Use Health Facility for Delivery	P-value	Use Post Natal Services	P-value
**Autonomy of Women**							
Not involved in decision-making	7627	9.1	< 0.001	25.0	< 0.001	12.3	< 0.001
Involved in decision-making	12,137	26.2		50.3		24.9	
**Age Group**							
< 25	4768	12.2	< 0.001	32.1	< 0.001	16.5	< 0.001
25–34	9529	21.9		43.6		21.2	
35+	5467	22.1		42.4		21.2	
**Level of Education**							
No Formal Form Education	9356	4.1	< 0.001	14.8	< 0.001	11.0	< 0.001
Primary Level of Education	2852	18.5		41.7		23.3	
Secondary Level of Education	5848	35.0		67.0		29.6	
Post Level of Secondary	1708	53.5		89.1		31.5	
**Religion**							
Christianity	6997	37.9	< 0.001	68.7	< 0.001	30.6	< 0.001
Islam	12,767	9.6		25.1		14.3	
Wealth Index***							
Poor	8976	5.5	< 0.001	16.5	< 0.001	13.3	< 0.001
Middle	3980	16.3		41.2		19.8	
Rich	6808	40.1		71.8		29.2	
Residence***							
Urban	7511	35.4	< 0.001	63.4	< 0.001	28.3	< 0.001
Rural	12,253	9.9		26.5		15.0	
Region***							
North Central	2812	14.1	< 0.001	50.5	< 0.001	18.1	< 0.001
North East	3611	3.8		25.5		18.0	
North West	7396	4.1		16.0		10.0	
South East	1815	38.1		82.5		46.2	
South South	1534	43.4		55.8		22.9	
South West	2595	64.7		82.1		33.8	
Ethnicity***							
Hausa/Fulani	9849	4.2	< 0.001	16.9	< 0.001	12.2	< 0.001
Igbo	2353	44.5		82.9		41.6	
Yoruba	2218	62.6		81.9		35.2	
Others	5343	19.2		48.2		18.8	
CEB***							
One	3076	25.6	< 0.001	52.0	< 0.001	22.3	< 0.001
Two	3549	27.1		48.5		22.1	
Three	3019	24.4		47.1		21.3	
Four	10,120	13.7		32.3		18.3	
Partners’ Age Group***							
< 25	357	15.2	< 0.001	33.2	< 0.001	20.7	= 0.003
25–34	5238	19.4		40.8		21.1	
35–44	7908	23.4		46.2		20.6	
45+	6260	15.3		33.5		18.5	
Partners’ Level of Education***							
No Formal Form Education	7554	4.4	< 0.001	13.1	< 0.001	9.5	< 0.001
Primary Level of Education	2726	17.5		39.77		23.8	
Secondary Level of Education	6499	29.6		58.5		26.9	
Post Level of Secondary	2985	38.3				28.7	

***Significant at p < 0.001

Table 4 summarizes the findings from the analysis of the autonomy of women and maternal health-care services utilization using logistic regression models. There was a positive association between maternal healthcare services utilization and the autonomy of women. When compared to women who did not participate in family decision-making, those who participated in household or family decision-making were more than two times more likely to use maternal health-care services.

**Table 4:** Logistic regression models assessing the odds of maternal health-care services utilization

Variables	ANC Visits		Health Facility Delivery		Post-natal Services	
	OR	95% CI	OR	95% CI	OR	95% CI
**Autonomy of Women**						
Not involved in household decision-making	1.00		1.00		1.00	
Involved in household decision-making	3.57***	3.11–4.10	3.03***	2.75–3.33	2.36***	2.11–2.64

***Significant at p < 0.001

Having adjusted for socio-demographic and other selected variables in Table 5, the autonomy of women ((ANC visits (OR 1.24; 95% CI 1.06–1.44)), (health facility delivery (OR 1.19; 95% CI 1.08–1.31)) and (postnatal services (OR 1.49; 95% CI 1.32–1.68))) maintained a positive association with the utilization of maternal health-care services. When compared to women who did not participate in household or family decision-making, women who participated in household or family decision-making were more likely to use maternal health-care services. In addition, model 5 revealed that mothers with primary education ((ANC visits (OR 1.56; 95% CI 1.29–1.90)), (health facility delivery (OR 1.34; 95% CI 1.16–1.54)) and (postnatal services (OR 1.29; 95% CI 1.10–1.51))) and secondary education ((ANC visits (OR 1.80; 95% CI 1.48–2.20)) and (health facility delivery (OR 1.77; 95% CI 1.51–2.07)) and (postnatal services (OR 1.30; 95% CI 1.10–1.54))) were more likely to use maternal health-care services than mothers with no formal form of education. When compared to mothers in urban regions, the likelihood of maternal health-care services utilization was shown to be lower in rural areas ((ANC visits (OR 0.68; 95% CI 0.59–0.78)), (health facility delivery (OR 0.74; 95% CI 0.64–0.86)) and (postnatal services (OR 0.81; 95% CI 0.71–0.93))). When compared to mothers of Hausa/Fulani descent, mothers of the Igbo tribe ((ANC visits (OR 3.70; 95% CI 0.2.81–4.86)), (health facility delivery (OR 6.29; 95% CI 4.85–8.15)) and (postnatal services (OR 2.39; 95% CI 1.91–3.01))) and mothers of the Yoruba tribe ((ANC visits (OR 9.66; 95% CI 7.55–12.37)), (health facility delivery (OR 5.84; 95% CI 4.55–7.49)) and (postnatal services (OR 1.95; 95% CI 1.57–2.42))) reported higher odds of maternity health-care services utilization. In addition, the higher the level of educational attainment, the higher the use of maternal health-care services. Women who claimed their husbands/partners had secondary education (ANC visits (OR 1.34; 95% CI 1.07–1.68)), (health facility delivery (OR 1.81; 95% CI 1.55–2.11)) and (postnatal services (OR 1.85; 95% CI 1.55–2.20))), and post-secondary education (ANC visits (OR 1.65; 95% CI 1.25–2.19)), (health facility delivery (OR 2.48; 95% CI 2.04–3.02)) and (postnatal services (OR 2.23; 95% CI 1.83–2.72))) had a higher likelihood of maternal health-care services usage than husbands/partners of mothers with no formal form education.

**Table 5:** Logistic regression models assessing the odds of maternal health-care services utilization

Variables	ANC Visits		Health Facility Delivery		Postnatal Services	
	OR	95% CI	OR	95% CI	OR	95% CI
**Autonomy of Women**						
Not involved in household decision-making	1.00		1.00		1.00	
Involved in household household decision-making	1.24**	1.06–1.44	1.19**	1.08–1.31	1.49***	1.32–1.68
**Age Group**						
< 25	1.00		1.00		1.00	
25–34	1.27**	1.08–1.50	1.06	0.92–1.22	1.10	0.96–1.27
35+	1.54***	1.22–1.93	1.20	0.99–1.45	1.09	0.90–1.32
**Level of Education**						
No Formal Form of Education	1.00		1.00		1.00	
Primary Level of Education	1.56***	1.29–1.90	1.34***	1.16–1.54	1.29**	1.10–1.51
Secondary Level of Education	1.80***	1.48–2.20	1.77***	1.51–2.07	1.30**	1.10–1.54
Post Secondary Level of Education	2.30***	1.74–3.03	4.21***	3.19–5.55	1.12	0.90–1.41
**Religion**						
Christianity	1.00		1.00		1.00	
Islam	0.60***	0.49–0.73	0.85	0.72–1.02	0.89	0.76–1.04
**Wealth Index**						
Poor	1.00		1.00		1.00	
Middle	1.42***	1.19–1.68	1.71***	1.48–1.98	0.91	0.78–1.05
Rich	1.97***	1.65–2.35	2.49***	2.10–2.94	0.90	0.76–1.07
**Residence**						
Urban	1.00		1.00		1.00	
Rural	0.68***	0.59–0.78	0.74***	0.64–0.86	0.81**	0.71–0.93
**Ethnicity**						
Hausa/Fulani	1.00		1.00		1.00	
Igbo	3.70***	2.81–4.86	6.29***	4.85–8.15	2.39***	1.91–3.01
Yoruba	9.66***	7.55–12.37	5.84**	4.55–7.49	1.95***	1.57–2.42
Others	2.12***	1.67–2.69	2.44***	2.03–2.91	1.03	0.86–1.23
**CEB**						
One	1.00		1.00		1.00	
Two	0.92	0.78–1.09	0.66***	0.55–0.78	0.97	0.83–1.12
Three	0.79*	0.65–0.95	0.63***	0.53–0.75	0.95	0.81–1.11
Four	0.61**	0.50–0.74	0.54***	0.45–0.64	1.04	0.89–1.21
**Partners’ Age Group**						
< 25	1.00		1.00		1.00	
25–34	1.25	0.79–1.98	1.45	0.98–2.15	0.89	0.59–1.35
35–44	1.40	0.87–2.24	1.81**	1.22–2.69	0.76	0.49–1.17
45+	1.30	0.79–2.12	1.57*	1.05–2.34	0.82	0.52–1.28
**Partners’ Level of Education**						
No Formal Form Education	1.00		1.00		1.00	
Primary Level of Education	1.14	0.89–1.44	1.46***	1.24–1.72	1.76***	1.47–2.11
Secondary Level of Education	1.34*	1.07–1.68	1.81***	1.55–2.11	1.85***	1.55–2.20
Post Secondary Level of Education	1.65***	1.25–2.19	2.48***	2.04–3.02	2.23***	1.83–2.72

***Significant at p < 0.001, **=p < 0.01 and *=p < 0.05

## Discussions

The purpose of this study was to investigate the influence of the autonomy of women on maternal health-care services utilization in Nigeria. Maternal health-care services utilization is a composite index of 8 or more ANC visitations, delivery at the health facility, and postnatal care for a current or recent childbirth in the five-year preceding the current Nigeria Demographic and Health Survey. The composite index of women’s decision-making autonomy on health-care for respondents, large household purchases, and visits to relatives or family were used to measure women’s autonomy. A weighted sample of 19,764 married women who gave birth in the five-year preceding the survey was used [8]. The findings, as corroborated by other studies [10, 46], revealed the autonomy of women had a significant influence on maternal healthcare services utilization in Nigeria. The findings also revealed low maternal health-care services utilization [16, 47]. In Nigeria, the potential consequence of low usage of healthcare services among women might be high maternal and child mortality [10].

This highlights the importance of intervention programmes and policies that will increase the autonomy of women to improve the utilization of maternal health-care services in Nigeria. Women of high autonomy are more likely to use health-care facilities during pregnancies, deliveries and postnatal care periods compared to women of low autonomy [8]. There is a link between the autonomy of women and maternal health-care services utilization. Higher women’s autonomy in decision-making on health-care services will increase the use of maternal health-care services and consequently reduce pregnancy risks, complications, and childhood and maternal mortality. It will lead to better pregnancy outcomes, welfare of children and mothers [8, 48].

The findings also revealed that women’s education was related to their use of maternal health-care [46, 47, 49]. As women’s education levels rise, so does their use of maternal healthcare services. Education improves women’s access to healthcare facilities [8] as well as their ability to participate in family decision-making [50]. School also empowers and boosts women’s self-esteem [50]. The level of education of husbands/partners yielded similar results. Women with educated spouses were more likely to use maternal healthcare services than their counterparts. Educated men will appreciate women more and understand why they should be involved in family decisions.

Muslims used maternal healthcare services less frequently than Christians. This could be related to the fact that Christianity is more open to western education, which encourages women’s autonomy and the use of maternal health-care services. Household wealth influenced the use of maternal healthcare services in Nigeria, according to the study. Women from wealthy households used maternal health-care services more frequently [46, 49]. Many women’s inability may be due to a lack of financial resources.

Furthermore, women in urban areas used maternal healthcare services more than women in rural areas [47]. Women in cities have access to more health and education services. In comparison to rural women, they are more empowered and liberal. Women in urban areas may be more likely to use maternal health facilities because of this. The study discovered disparities in maternal healthcare service utilization among Nigeria’s three major ethnic groups. In Nigeria, the Yoruba tribe is the most educated and sociable, followed by the Igbo tribe. Hence, more use of maternal health-care services by the Yoruba and Igbo tribes compared to the Hausas.

Furthermore, as the number of children increased, the usage of maternal healthcare services declined. As the number of children grows, so does the cost of their care. This could explain why women with fewer children use maternal healthcare services more frequently.

## Conclusion

In Nigeria, maternal health-care services utilization is linked to women’s autonomy. As a result, women’s autonomy as a predictor should be examined alongside many other determinants to encourage and enhance the utilization of maternal health services. Increased utilization of maternal health services in Nigeria could be aided by effective policies, programmes, and interventions concentrating on increasing the autonomy of women.

## Strengths and limitations

The survey is nationally representative, allowing the findings to be generalized across Nigeria. The study, however, had some flaws. Few household’s decision-making indexes may not adequately capture women’s autonomy [8]. The cause-effect linkages cannot be deduced due to the cross-sectional character of the survey, hence interpretations of findings should be done with caution. Due to the self-reported nature of the data, the survey may suffer from recall bias and social desirability [18, 51].

## Data Availability

Data were sourced from Demographic and Health survey (DHS) and are available here: http://dhsprogram.com/data/available-datasets.cfm.
